# Safety of G2-S16 Polyanionic Carbosilane Dendrimer as Possible HIV-1 Vaginal Microbicide

**DOI:** 10.3390/ijms23052565

**Published:** 2022-02-25

**Authors:** Alba Martin-Moreno, Rafael Ceña-Diez, María Jesús Serramía, José Luis Jiménez, Rafael Gómez-Ramírez, Mariángeles Muñoz-Fernández

**Affiliations:** 1 Sección Inmunología, Laboratorio InmunoBiología Molecular, Hospital General Universitario Gregorio Marañón, 28007 Madrid, Spain; albamartinm35@gmail.com (A.M.-M.); rcena48@gmail.com (R.C.-D.); mariajesus.serramia@salud.madrid.org (M.J.S.); 2 Laboratorio de Inmunobiología Molecular, Instituto de Investigación Sanitaria Gregorio Marañón (IISGM), 28007 Madrid, Spain; joseluis.jimenez@salud.madrid.org; 3 Spanish HIV HGM BioBank, 28007 Madrid, Spain; 4 Networking Research Center on Bioengineering, Biomaterials and Nanomedicine (CIBER BBN), 28029 Madrid, Spain; rafael.gomez@uah.es

**Keywords:** HIV-1 infection, G2-S16 dendrimer, topical vaginal application, organ tissue, mice

## Abstract

The UNAIDS objective for 2020 was 500,000 new HIV-1 infections per year; however, the latest annual reported data confirmed 1.7 million new HIV-1 infections in that year. Those data evidences the need for new prevention strategies and prophylactic treatments. This prevention crisis occurred in spite of the knowledge and availability of efficient prevention strategies. The G2-S16 is a microbicidal polyanionic carbosilane dendrimer currently being tested for topical vaginal application, which has been shown to be efficient in the prevention of HIV-1 infection. However, safety tests were lacked. For this purpose, we injected intravenously G2-S16 dendrimer to CD1 mice, thereby analyzing the hemogram, blood biochemical markers of systemic damage, accumulation in the organs and organ-tissue damage in heart, spleen, kidney, liver and brain. This work shows that even if the G2-S16 dendrimer penetrates the epithelial tissue, it does not cause vaginal irritation or tissue damage. Moreover, the i.v. injection of the G2-S16 dendrimer did not cause a damaging effect on the studied organs and it did not modify the hemogram or the biochemical plasma markers. In conclusion, the G2-S16 dendrimer has a very good safety profile, indicating that this molecule can be a very safe and efficient vaginal microbicide.

## 1. Introduction

Entry inhibitors are ideal candidates for the development of an effective microbicide due to their ability to prevent viral entry which is the first step in viral infection. Among most common drug, in terms of entry inhibitors, is maraviroc (MVC), which is a CCR5 receptor antagonist. Currently, an attachment inhibitor (fostemsavir), a post-attachment inhibitor (ibalizumab), the fusion inhibitors enfuvirtide and the reverse transcriptase inhibitor tenofovir (TFV) have been approved for use in HIV patients. However, these drugs are presently seldom used in clinical care. Unfortunately, when tested as microbicides in clinical trials, many vaginal microbicide formulations based on entry inhibitors failed to cause a protective effect due to their lack of efficacy and their unsuitable formulation, which led to irritability and inflammation [1,2,3]. Additionally, as happens with other drugs, entry inhibitors are affected by drug resistance mutations and could be affected by viral tropism (e.g., CCR5 antagonists) and subtype. Altogether, a need for new categories of antiretroviral drugs which could act as microbicides has emerged.

Additionally, a failure of the available preventive methods is highly correlated with poverty, child marriage, and gender-based or partner violence [4]. These factors leave women in a vulnerable position and less able to protect themselves against infection by HIV-1 and other different viruses. There is an urgent need to develop novel strategies to address the issue, since these factors leave women in a vulnerable position and makes them less able to protect themselves against HIV-1 infection. However, a cheap topical microbicide against HIV-1 could be easily used by women, independently of their cultural and social situation, and with or without the control and consent of men [5]. However, there are currently no microbicides available that can provide protection against HIV-1 infection [6]. Preventive topical microbicides are designed to be applied vaginally or rectally to provide protection before, during, and after sexual intercourse [3,7]. The microbicide should prevent infection without damaging vaginal or rectal tissues or without causing systemic damage, should it reach the bloodstream. Moreover, it should not interfere with the normal function of the local immune system against other pathogens or non-infectious diseases [8]. Researchers have been trying to design efficient options and several drug combinations have reached clinical trials but have so far failed because of unexpected toxicity or lack of efficacy [1,2].

The G2-S16 dendrimer is a water-soluble polyanionic carbosilane dendrimer, selected as a potential vaginal topical microbicide based on its simplicity, versatility, flexibility, moderate reaction conditions, short reaction times, wide availability of reagents, high reproducibility and quantitative yields of reaction. The molecule has been synthesized as topical gel formulation from the G2-S16 dendrimer solution of 3% (weight/volume) in a gel based on HEC 2%. The G2-S16 dendrimer proved to be safe and effective against HIV-1 as a topical vaginal microbicide in animal models. Previously, the G2-S16 dendrimer was shown to be harmless and effective in the prevention of HIV-1 infection in different in vitro, ex vivo, and in vivo models [9,10,11,12,13,14,15]. The G2-S16 dendrimer acts as an entry inhibitor binding to HIV-1 gp120 [16] and, it is also noteworthy that HIV-1 does not develop resistance against G2-S16 dendrimer [17].

To further guarantee the safety of the G2-S16 dendrimer as a microbicide, we used a murine model to study the in vivo biodistribution, anatomopathological impact and biochemical blood composition after systemic administration. In search for signs of potential tissue irritation or inflammation, we examined the histology of the vaginal epithelium in mice after exposure to G2-S16 dendrimer. We also studied potentially damaging systemic side-effects on the brain, heart, kidney, liver, and spleen. The data demonstrate that the G2-S16 dendrimer is not only an efficient prophylactic compound against HIV-1 infection, but it is also safe for use as a topical vaginal microbicide, suggesting its possible use as a microbicide in a clinical setting.

## 2. Results

### 2.1. In Vivo Imaging of G2-S16-FITC Dendrimer in the Vagina of BALB/c Mice

Previous studies showed that the G2-S16 dendrimer does not modify the immune barrier of the female reproductive tract, and it is an effective inhibitor of HIV-1 infection [14]. Therefore, we aimed to study its biocompatibility in mice. In order to be able to visualize the G2-S16 dendrimer in the tissue, an FITC-labelled G2-S16 dendrimer was used. In previously published studies performed in mice, the G2-S16 dendrimer was applied at 3% *w*/*v*, and it was able to prevent HIV infection in BLT humanized mice [18]. Since previously published data indicated that the toxicity of FITC-labelled G2-S16 dendrimer was 5-to-10 times more cytotoxic than G2-S16 dendrimer, the G2-S16 FITC concentration used for visualization was 10 times smaller (3% vs. 0.3%) to ensure no FITC damage generation.

**Figure 1:**
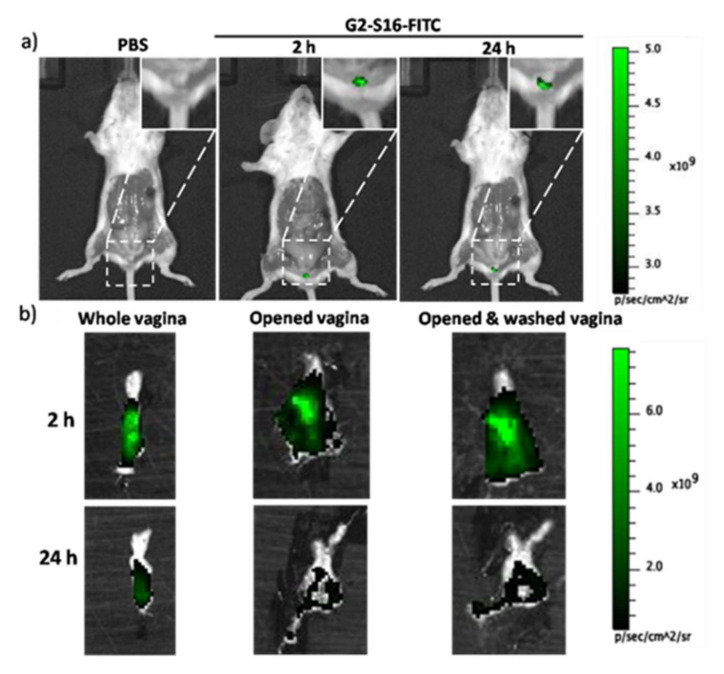
In vivo imaging of G2-S16-FITC dendrimer in the vagina of BALB/c mice. PBS control or G2-S16-FITC dendrimer was vaginally applied, and images of (**a**) whole mice and (**b**) extracted, opened and PBS-washed vaginas 2 h and 24 h after treatment application.

We wondered if, upon topical application in the mouse vagina, G2-S16 dendrimer remains in the lumen of the vagina or is able to cross the vaginal epithelium, and to penetrate the tissue. In order to evaluate the dendrimer’s ability to penetrate the vaginal epithelium, we treated BALB/c mice with either 1% PBS or the combination of 0.3% *w*/*v* G2-S16-FITC and 2.7% *w*/*v* G2-S16 dendrimers, and visualized the tissue fluorescence using an IVIS Lumina Image System as indicated in Materials and Methods. We determined the fluorescence at 2 h and 24 h post-treatment in the whole mouse (Figure 1a) and in the open vagina before and after washing with PBS to clean the remaining G2-S16 dendrimer from the lumen (Figure 1b). At 2 h post treatment, we detected intense fluorescence in the vagina that remained after washing. However, the fluorescence faded after 24 h (Figure 1), which suggested that G2-S16-FITC dendrimer can cross the vaginal epithelium; however, it is cleared out by the body.

### 2.2. Confocal Microscopy of G2-S16-FITC Dendrimer in the Vagina of BALB/c Mice

To further assess the penetration and permanence of the G2-S16 dendrimer in the vaginal tissue, extracted vaginas were stained and observed under the confocal microscope. Mice were divided in a PBS control group and combined-treated group (*w*/*v* G2-S16-FITC/G2-S16 dendrimer 9:1). Mice were sacrificed at 2 h and 24 h after treatment. Additionally, another group of mice were treated with a daily application of the combination for seven days and were sacrificed 24 h after their last application, to study the potential accumulation of the G2-S16 dendrimer. We analyzed tissue samples from the vagina, fornix and ectocervix, which are areas of the female reproductive tract that the treatment would reach, and stained them with CD192-Alexa Flour 647 and DAPI to be able to distinguish the tissue architecture. The epithelium of the vagina and ectocervix is a stratified squamous epithelium, whereas the endocervix is characterized by a simple columnar epithelium.

**Figure 2:**
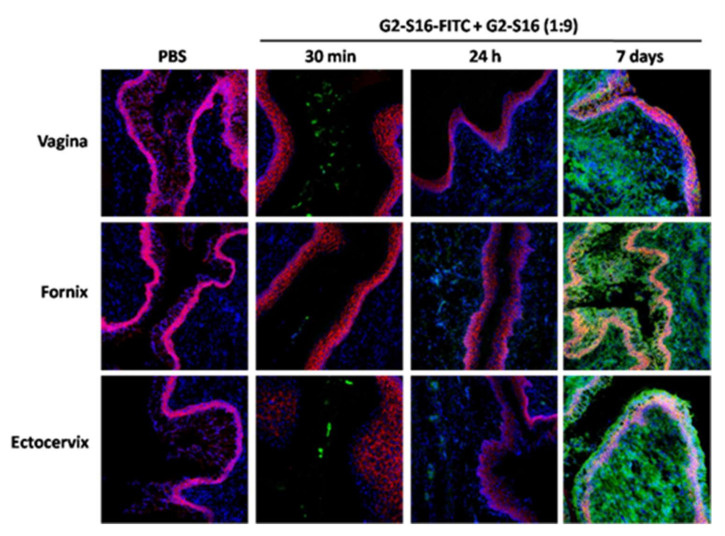
Accumulation of G2-S16-FITC dendrimer in the vagina, fornix and ectocervix of BALB/c mice. PBS control or G2-S16-FITC and G2-S16 dendrimers (at 1:9 rate) were applied in the vagina mice, and tissue samples of vagina, fornix and ectocervix were observed at 30 min, 24 h and 7 daily applications. Samples were stained with CD192-Alexa Flour 647 and DAPI.

In all three of the areas studies, we observed fluorescence from G2-S16-FITC dendrimer in the lumen 30 min after application of the treatment, being lighter in the fornix (Figure 2). In agreement with the imaging results, the G2-S16-FITC dendrimer penetrated the mucosa and submucosa, but the fluorescent signal was very dim 24 h after treatment (Figure 2). However, seven days of daily application resulted in a very intense signal in the mucosa and submucosa of all vagina, fornix, and ectocervix (Figure 2), thus proving that G2-S16-FITC dendrimer crosses the epithelial barrier and penetrates the tissue, and suggesting that the clearance of the G2-S16-FITC dendrimer is not as efficient as previously thought, raising possible toxicity or tissue-damage issues.

**Figure 3:**
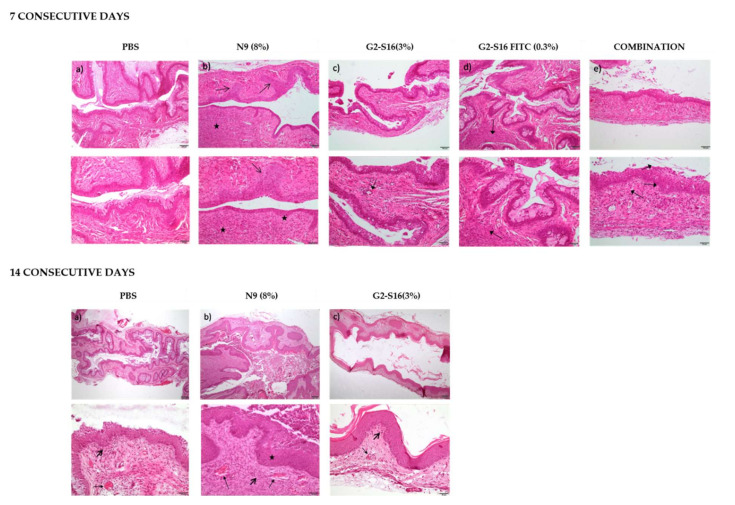
Histological imagens of vaginal tissue after 7 or 14 days of consecutive treatment with G2-S16 dendrimer. **Upper panel.** BALB/c mice were treated with 7 daily applications of (**a**) PBS, (**b**) N9, (**c**) G2-S16 dendrimer (3% *w*/*v*), (**d**) G2-S16-FITC dendrimer (0.3% *w*/*v*) or (**e**) a combination of G2-S16 dendrimer (2.7% *w*/*v*) and G2-S16-FITC dendrimer (0.3% *w*/*v*). Epithelial hyperplasia (arrows) and inflammatory infiltration and fibrosis (star) were found in the samples treated with N9 (**b**). The samples from mice treated with the G2-S16 dendrimer (**c**–**e**) presented some inflammatory infiltration (small arrows). The images on the bottom are zoom-ins at the images on the top. **Lower panel.** BALB/c mice were treated with 14 daily applications of (**a**) PBS, (**b**) N9 or (**c**) G2-S16 dendrimer (3% *w*/*v*). We observed different degrees of vascular congestion (small arrows), inflammatory infiltration (big arrows) and epithelial hyperplasia (*). The images on the bottom are zoom-ins at the images on the top.

**Figure 4:**
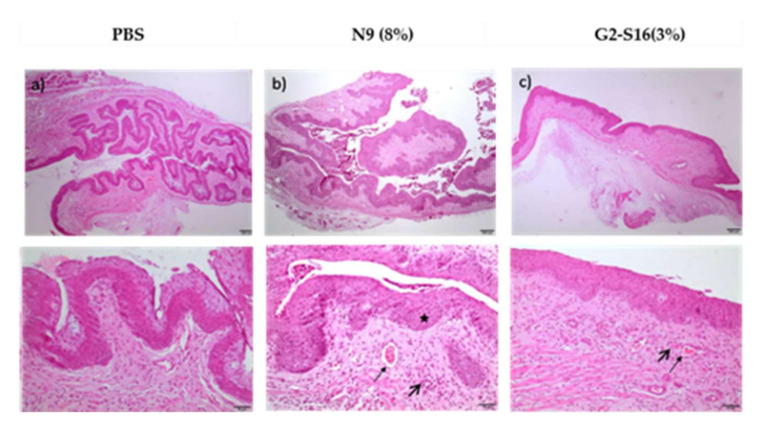
Histological images of CD1 mice vaginal tissue after 14-treatment with G2-S16 dendrimer. CD1 mice were treated with 14 daily applications of (**a**) PBS, (**b**) N9 or (**c**) G2-S16 dendrimer (3% *w*/*v*). We observed different degrees of vascular congestion (small arrows), inflammatory infiltration (big arrows) and epithelial hyperplasia (star). The images on the bottom are zoom-ins at the images on the top.

To investigate the hypothesis of whether the G2-S16 dendrimer accumulating in the vagina could cause any tissue damage or irritation, we performed an in vivo irritation assay in two different mice strains, namely BALB/c and CD1 mice. Twelve female mice of each strain were divided in 5 groups: 2 mice treated with PBS as negative control group, 2 mice treated with nonoxynol-9 (N9) a known compound causing vaginal irritation as control, 2 mice treated with G2-S16 dendrimer (3% *w*/*v*), 3 mice treated with G2-S16-FITC dendrimer (0.3% *w*/*v*), and 3 mice treated with the combination (0.3 *w*/*v* G2-S16-FITC and 2.7% *w*/*v* G2-S16 dendrimers). Mice were sacrificed after 7 or 14 days of daily treatment in the case of Balb-c (Table 1; Figure 3) and only at day 14 in the case of CD1 (Table 2; Figure 4), and the vaginas were extracted and processed for a histological evaluation of the tissue damage.

In order to quantify the effect of the treatment, a score (1–4) was assigned to each lesion type in each sample depending on the severity of the lesion. The scores in each sample were summed up to obtain a final score of tissue damage, which was interpreted as, minimum 1–4, average 5–8, moderate 9–11 and severe 12–16 [9] (Table 1 and Table 2). The results suggest that even if G2-S16 dendrimer accumulates in the tissues, it causes minimal vaginal irritation, and labeling with FITC increases the damage, but still under the levels considered minimum as in contrast to N9 taken as a control for its known toxicity.

Data showed that the effects of G2-S16 dendrimer on the vagina of BALB/c mice after 14 days of treatment are very similar to that described after 7 days of application. The tissue from all five mice treated with the G2-S16 dendrimer obtained a score considered as minimal damage. On CD1 mice, the results showed that G2-S16 dendrimer is slightly more harmful to CD1 mice than for BALB/c mice, although the tissue damage found is still considered of minimum severity, very close to the PBS control (Table 1). As expected, N9 presented toxicity levels between moderate and severe on both mice strains. In summary, these results demonstrate that the G2-S16 dendrimer (3% *w*/*v*) does not harm the tissue integrity of the vagina after 14 daily applications.

### 2.3. G2-S16 Dendrimer Does Not Modify the Hemogram of Mice or Plasma Biochemical Parameters after 7 or 14 Days of Vaginal Application

To study the effect of vaginally applied G2-S16 dendrimer on both the hemogram and biochemical plasma parameters, CD-1 mice were used due to their larger blood volume than BALB/c mice. Following either 7 or 14 days consecutive G2-S16 dendrimer vaginal administration, no significant changes for the untreated animals in any hemogram parameter was observed, including in the hematocrit, total white blood cells number, and total platelet count. Moreover, no significant changes in several plasma biochemical parameters used as biomarkers for kidney (creatinine, urea, phosphorus), lipid metabolism (cholesterol), or hepatic (alanine aminotransferase, alkaline phosphatase) function were observed when compared to untreated animals. Finally, protein levels (total, albumin and globulin) were also similar between PBS or G2-S16 dendrimer-treated groups. These results suggest that vaginally applied G2-S16 dendrimer has no systemic effects and does not cause organ-function damage after 14 days of vaginal application.

### 2.4. Mouse Survival after G2-S16 Dendrimer Intravenous Application

Our results suggest that G2-S16 dendrimer is safe for vaginal application considering the observed biocompatibility and lack of tissue and systemic damage. To confirm this conclusion, we wondered if a higher blood concentration of the G2-S16 dendrimer could have more significant systemic consequences with the objective of identifying the largest non-lethal G2-S16 dendrimer concentration, so as to use it in the following experiments.

**Figure 5:**
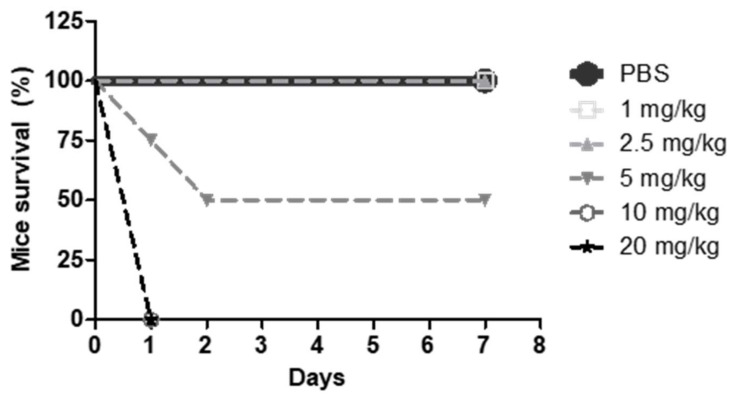
CD1 mice survival after i.v. application of G2-S16 dendrimer. CD1 mice were injected with G2-S16 dendrimer at different concentrations, and PBS as innocuous control. Survival of the mice after application is plotted as percentage of treated mice alive at each time point. Each group was formed by 4 animals.

To this end, CD1 mice were divided in six groups, treated intravenously for seven consecutive days with PBS or different doses of G2-S16 dendrimer (1, 2.5, 5, 10, and 20 mg/kg). The CD1 mice survival after treatment was noted (Figure 5) and 2.5 mg/kg of G2-S16 dendrimer was determined as the maximum non-lethal intravenous dose. As can be observed in Figure 5, i.v. administration of doses above 5 mg/kg caused the death of all animals in 24 h.

### 2.5. Effect of G2-S16 Dendrimer Intravenous Application on Hemogram and Biochemical Plasma Composition

We selected 1 and 2.5 mg/kg intravenous doses of G2-S16 dendrimer, to study the potential systemic effects of these doses of G2-S16 dendrimer that were the maximal doses that did not cause the death of any animal. The CD1 mice were treated intravenously for 7 consecutive days with PBS or G2-S16 dendrimer (1 or 2.5 mg/kg) and hemogram, and plasma biochemical parameters were analyzed after the treatment. Only an increase in total platelets and platelecrit was observed at the dose of 1 mg/kg, which was not observed at 2.5 mg/kg. No differences were found in any other measured hematocrit parameter when comparing G2-S16 dendrimer treated mice with PBS-treated mice (Table S1). Additionally, we measured blood biochemical markers from the same CD1 mice treated with PBS or G2-S16 dendrimer (1 or 2.5 mg/kg) in order to assess G2-S16-mediated potential damage (Table S2). Moreover, no significant differences were found between the PBS-treated and G2-S16 dendrimer-treated in biomarkers for kidney (creatinine, urea and phosphorous), pancreatic (lipase), hepatic (alanine aminotransferase, ALT; alkaline phosphatase, ALKP) function, or in cholesterol or protein levels. However, a statistically significant decrease in the G2-S16 dendrimer treated animals in both alanine aminotransferase (ALT) and bilirubin levels were observed. Further studies are required to determine the relevance of this finding. Taken together, these data indicate that G2-S16 dendrimer is biocompatible and safe.

### 2.6. G2-S16-FITC Biodistribution Dendrimer in Heart, Spleen, Kidney, Liver, and Brain Tissue

**Figure 6:**
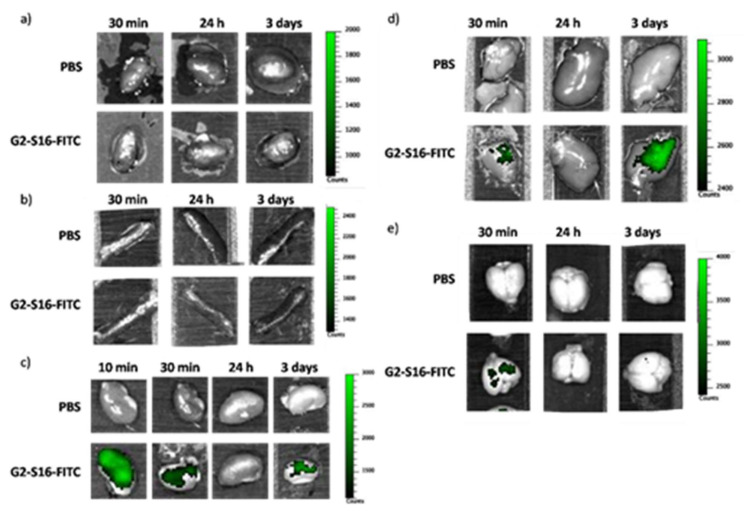
In vivo imaging of G2-S16-FITC dendrimer in different organs after IV-treatment of CD1 mice. CD1 mice were treated intravenously with PBS or G2-S16-FITC dendrimer and organs were harvested 30 min or 24 h after treatment, or 24 h after 3 daily applications. In vivo imaging was used to assess whether the G2-S16 dendrimer reaches and accumulates in the (**a**) heart, (**b**) spleen, (**c**) kidney, (**d**) liver and (**e**) brain. Intensity of fluorescence is represented in a scale of green.

To study dendrimer biodistribution, CD1 mice were i.v. injected with a single dose of florescence-labelled G2-S16-FITC (1 mg/kg), euthanized and heart, spleen, kidney, liver and brain extracted 30 min or 24 h after treatment. Then, images of the organs were obtained using an IVIS system as indicated in Materials and Methods. In other set of experiments, mice received daily i.v. doses of G2-S16-FITC (1 mg/kg) for 3 days. Then, they were sacrificed and heart, spleen, kidney, liver and brain extracted and fluorescence measured as indicated above. No fluorescence was observed in heart (Figure 6a) or spleen (Figure 6b) at any time point, suggesting that the G2-S16 dendrimer does not penetrate into these organs. However, in the case of kidney (Figure 6c) fluorescence was found at 30 min disappearing at 24 h after single dose. Since it was expected that kidney would be responsible for clearing G2-S16 dendrimer from the circulation, we studied what would happen at 10 min in this organ following an i.v dendrimer injection. We found an intense signal, larger than that found at 30 min, suggesting a fast clearance of the G2-S16 dendrimer by the kidney. Interestingly, in kidneys from animals receiving three daily doses of dendrimer, a significant fluorescence was observed after sacrifice, suggesting a dendrimer accumulation in the kidney that requires longer periods to be cleared. However, following G2-S16 treatment, no changes in plasma creatinine or urea were observed after 7 daily doses of dendrimer (Table S2). In the case of liver, very little accumulation was observed after 30 min following single i.v. administration that disappeared at 24 h (Figure 6d). Following three daily dendrimer i.v. administrations, a significative accumulation in the liver could be observed, although no increase in the plasma biomarkers of hepatic damage (AL T, ALKP) were observed after seven daily i.v dendrimer injections (Table S2). There is a very small accumulation of fluorescence in the brain 30 min after a single injection (Figure 6e) that disappeared at 24 h and that is absent following 3 daily i.v. dendrimer administrations. This would suggest that the G2-S16 dendrimer accumulation in the brain of any is minimal.

### 2.7. G2-S16 Dendrimer Causes no Histological Lesions in Heart, Spleen, Kidney, Liver, or Brain

In view of the imaging results that showed G2-S16-FITC dendrimer reaches and accumulates in some organs after being applied intravenously, we wondered if G2-S16 dendrimer could cause tissue damage. With the aim of studying the potential histological damage, we processed mice-treated heart, spleen, kidney, liver and brain for histological evaluation. Ten mice were divided in three groups and treated with 7 daily intravenous applications of PBS (3 mice), 1 mg/kg G2-S16 dendrimer (3 mice), or 2.5 mg/kg G2-S16 dendrimer (4 mice). The mice were sacrificed after 7 days of treatment, and the organs were extracted and processed for histological analysis. Each tissue was assigned a score depending on the severity as mentioned above.

**Figure 7:**
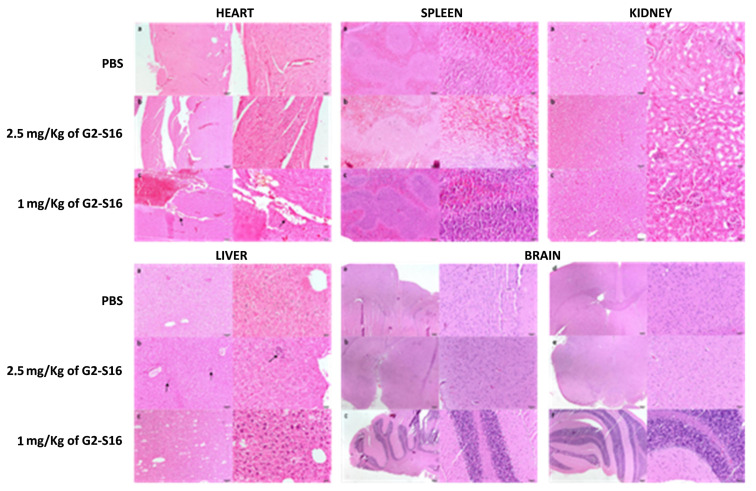
G2-S16 dendrimer causes no histological lesions in analyzed organs. Hearts from mice treated with PBS, 2.5 mg/kg of G2-S16 dendrimer or 1 mg/kg G2-S16 dendrimer were analyzed, and no relevant histological lesions were found in any of the samples, although the presence of adipocytes (arrows) in endocardium and myocardium were observed. Spleens and Kidneys from mice treated with PBS, 2.5 mg/kg of G2-S16 dendrimer or 1 mg/kg G2-S16 dendrimer were analyzed, and no histological lesions were found in any of the samples. Livers from mice treated with PBS, 2.5 mg/kg of G2-S16 dendrimer or 1 mg/kg G2-S16 dendrimer were analyzed. Although minimal histological lesions were found in some of the samples, including multifocal inflammatory infiltrates (arrows) and vacuolar degeneration, there was no significant difference between the PBS and the G2-S16 dendrimer-treated groups. Brains from mice treated with PBS, 2.5 mg/kg of G2-S16 dendrimer or 1 mg/kg G2-S16 dendrimer (images not shown) were analyzed, and no histological lesions were found in any of the samples. Images were obtained from midbrain, thalamus and cerebellum. The images on the right are zoom-ins at the images on the left.

As expected, given the lack (or minimal) dendrimer accumulation, a histological evaluation of the heart, brain, and spleen (Figure 7) showed no tissue damage in the organs from mice treated with 1 or 2.5 mg/kg of G2-S16 dendrimer. Similarly, the kidney (Figure 7) suffered no histological modifications besides the observed G2-S16 dendrimer accumulation over time. Although the liver was the most affected organ (Figure 7), as we found some minimal histological damage, the affects were minimal and there were no differences between the PBS controls and the G2-S16 dendrimer-treated mice.

## 3. Discussion

The availability of a topical microbicide against HIV-1 infection would offer an alternative to condoms as the most feasible method for HIV-1 prevention and would greatly contribute to a decrease in the new cases of HIV-1 infections worldwide and halt the epidemic [3]. In spite of the knowledge of successful HIV-1 prevention strategies, such as condom use, reduction of sexual partners, pre-exposure prophylaxis (PrEP) [19,20] and early diagnosis and treatment [21], HIV-1 continues to spread at an alarming rate, especially in developing countries, where women are a remarkably vulnerable. Microbicides are a potential HIV-1 prophylactic method that women can easily control and do not require the cooperation, consent or even knowledge of the partner, and thus, it would empower women in developing countries or resource-poor settings to protect themselves and their partners. There is no effective anti-HIV-1 microbicide available yet, although many have reached clinical trials.

Some non-specific surfactants, such as N9, SAVVY^®^ or C31G, were tested as potential microbicides, but were discarded due to their toxicity [22]. Similarly, their acid form caused mild to moderate vulvar irritation [23]. Carbopol^®^ 974P (BuffereGe 0.5%l) was well tolerated, but showed no significant prevention of HIV-1 vaginal transmission [24]. Some polyanionic polymers that blocked the binding of HIV-1 to target cells were also studied as potential microbicides, but they caused some disruption in the mucosal epithelial surface. Some examples of this family of microbicides include cellulose sulfate gel (Eshercell), carrageenan gel (Carraguard^®^) or naphthalene-sulfonate gel (PRO200). None of them were effective at halting heterosexual HIV-1 spread, and PRO200 even leads to an increased risk of HIV-1 infection [25]. Cellulose acetate phthalate-based microbicides caused heavy vaginal discharge in all recipients, which led to their elimination and thus to their failure as microbicides [26]. A more successful path has been followed by microbicides based on antiretrovirals (ARV) that specifically block the lifecycle of HIV-1. The main example is the reverse transcriptase inhibitor tenofovir (TFV), which is one of the most studied microbicides for topical vaginal application against HIV-1. The success of the first phase II clinical trial of an ARV-based microbicide CAPRISA 004 (Centre for the AIDS Program of Research in South Africa) was achieved using a TFV-based gel to prevent man-to-woman HIV-1 transmission.

This study was key in showing that ARV can be effective when used as topical microbicide, as it reduced HIV-1 acquisition by 39% if South African women, although vaginal microbiota modulated the efficacy of the topical microbicide in this trial. However, more recent failures of TFV as vaginal microbicide in clinical trials, such as VOICE study (Vaginal and Oral Interventions to Control the Epidemic) [27,28] demonstrate that there is still need for improvement and more efforts are needed to find effective microbicide strategies [27]. Another example of ARV candidate in the microbicide field is dapivirine (DPV), an antiretroviral that allosterically binds to HIV-1 reverse transcriptase and prevents HIV-1 infection, as it has showed a strong activity against various HIV-1 isolates in vitro [29]. The DPV has been tested in several application forms, including vaginal gels, films or intravaginal rings [29,30,31,32,33,34]. In spite of reaching phase II clinical trials, DPV has been shown to cause side effects such as inflammation, reddening or swelling of the cervix, urinary tract infection, loss of bladder control, headache, pain during sex, and pelvic pain [32,35,36] (Else, Taylor et al., 2011; Baeten, Palanee-Phillips et al., 2016; Nel, van Niekerk et al., 2016) [1,2,3]. A vaginal ring containing DPV is being tested in adolescent and young women [37]. Thus, the majority of topical microbicides failed due to a lack of efficacy, toxicity or adherence [25,38]. With the aim of filling the need of a feasible and accessible prophylactic option, the G2-S16 dendrimer emerges as a potential effective topical microbicide against HIV-1 infection. Previous studies have proven its efficiency against HIV-1 and HSV-2 in vitro and in vivo, even in the presence of semen [13], and have shown that it does not generate drug resistance [17], or affect the main agents of the immune system of the female reproductive tract [14].

Data suggest that G2-S16 dendrimer is safe as a microbicide since vaginal applications for either 7 or 14 consecutive days in mice did not modify either the hemogram or plasma concentration of several biochemical markers for kidney, liver, pancreas diseases or cholesterol or protein levels [39], thereby indicating that the dendrimer does not diffuse in significant amounts from the vagina to the blood stream or that if it does, it is not toxic. When high doses of the dendrimer were administered intravenously in different patterns, we found that the compound was biocompatible as indicated by the lack of changes in the hemogram of the animals and that the changes produced in plasma biochemical markers following i.v. administration, i.e., about 50% decrease in plasma ALT levels, does not indicate liver damage. On the contrary, low ALT plasma levels are considered healthy, and only very low levels of this enzyme, far from being reached by G2-S16 treatment, have been linked to increased long-term mortality. Something similar can be said for the observed decrease in bilirubin plasma concentration. Thus, these data indicate that liver is not damaged by G2-S16 dendrimer. However, more studies are needed to identify the mechanisms involved in these changes. The lack of toxicity of G2-S16 was further assessed by histological studies of heart, spleen, kidney, liver and brain that showed no sign of histological lesions, further confirming the biosafety of the G2-S16 dendrimer.

Biodistribution studies following the i.v. administration of fluorescence-labelled G2-S16 dendrimer showed a lack of significant incorporation into brain, heart or spleen. In the case of kidney, there was a very early accumulation 10 min after administration that quickly disappeared, suggesting that the dendrimer was rapidly cleared from the body by the kidney. However, when animals received three daily doses of the dendrimer, a significant fluorescence was observed, suggesting that dendrimer accumulation in the kidney requires longer times to be cleared. This did not lead to kidney toxicity since no increase in plasma markers (urea or creatinine) for kidney damage or histological damage were observed. In the case of liver, a marked accumulation in the liver could be observed after 3 daily i.v. dendrimer doses, although no increase in plasma biomarkers of hepatic damage (ALT, ALKP) or relevant histological damage were observed after 7 daily i.v dendrimer injections.

## 4. Materials and Methods

### 4.1. G2-S16 Dendrimer, Reagents and Antivirals

Water-soluble polyanionic carbosilane dendrimer G2-S16 (C_112_H_244_N_8_Na_16_O_48_S_16_Si_13_; MW = 3717.15 g/mol) is a second-generation dendrimer scaffold built from a silicon atom core which is fully capped on the surface with 16 sulfonate groups, as previously described [15]. Analogous G2-S16 FITC dendrimer (C_129_H_249_N_9_Na_14_O_47_S_15_Si_13_; MW = 3946.29 g/mol) were also synthesized as previously described [15]. Dendrimer dilutions were prepared in nuclease-free water (Promega, Madison, WI, USA). PBS (Thermofisher, Madrid, Spain) was used as innocuous control; medroxyprogesterone acetate (Depo-Provera^®^, New York, NY, USA) was used for synchronizing the estrous cycle of female mice; hydroxyethyl cellulose (HEC; NIH-ARRRP) gel (Sulky, Lab. Bohm, Fuenlabrada, Spain) as the vehicle for G2-S16 and G2-S16-FITC [40] dendrimers; nonoxynol-9 (N9, C_33_H_60_O_10_; Mw: 616.83 g/mol) as a positive control for vaginal irritation; isoflurane (C_3_H_2_ClF_5_O, MW: 184.5 g/mol, Forane^®^, Abbott, Peru) and ketamine/xylazine (C_13_H_16_NCLO; MW: 237.72 g/mol; C_12_H_16_N_2_S; MW: 220.33 g/mol) as anesthetic.

### 4.2. Mice Strains

The BALB/c and CD1 mice were purchased from Charles River Laboratories (Wilmington, MA, USA) and housed in the animal facility at the Centro de Biología Molecular Severo Ochoa (CBMSO, Madrid, Spain). The murine studies were approved by the CBMSO Institutional Animal Care and Ethical Committee (CEEA, CBMSO, Madrid, Spain) and all the experiments were performed following AEC-CBMSO and National Guidelines and Regulations (Real Decreto 1201/2005). Eight-week-old female BALB/c mice were used for the vaginal application assays. Female CD1 mice with a weight of 30 g were used for intravenous blood application analysis.

### 4.3. Vaginal Irritation Assay

A vaginal irritation assay was performed to evaluate the toxicity of G2-S16 and G2-S16-FITC dendrimers in BALB/c mice treated for 7 and 14 consecutive days and CD1 mice treated for 14 consecutive days. Negative control gel was formulated by mixing HEC gel (NIH-ARRRP) with sterile PBS to a final PBS concentration of 1%, and 8% (*w*/*v*). Nonoxinol-9 was added to the gel as an irritation agent control. The G2-S16 or G2-S16-FITC dendrimer were added to the 1% HEC gel to a final concentration of 3% or 0.3% *w*/*v*, respectively. Mice were injected subcutaneously with 2 mg of medroxyprogesterone acetate in 200 μL of PBS 5 days prior to treatment. All mice were randomly distributed in groups and treated with daily vaginal applications of 30 μL of treatment. Treatment was applied with a vaginal gavage needle in mice previously anesthetized with isoflurane. After treatment, mice were sacrificed and vaginas were extracted and conserved in 3.7–4% *w*/*v* formaldehyde.

### 4.4. Intravenous Application of G2-S16 and G2-S16-FITC Dendrimers in CD1 Mice

In order to study the systemic effects of the highest possible non-lethal blood concentration of G2-S16 and G2-S16-FITC dendrimers, the treatment was applied intravenously to CD1 mice. Both dendrimers were diluted in 100 μL of PBS at the indicated concentration for each mouse and G2-S16 dendrimer. Mice were immobilized and the treatment was injected via the tail vein.

### 4.5. In Vivo Imaging of the G2-S16-FITC Dendrimer

To study penetration in the vaginal tissue after vaginal application, BALB/c mice were injected subcutaneously with 2 mg medroxyprogesterone acetate 5 days prior to treatment. Mice were divided into two groups and treated with HEC gel with 1% PBS, or HEC gel with G2-S16 dendrimer (2.7% *w*/*v*) and G2-S16-FITC dendrimer (0.3% *w*/*v*) to final 3% *w*/*v* total dendrimer concentration. Images of the vagina were acquired 2 h and 24 h after treatment, and after PBS washed, to be able assess whether the G2-S16 dendrimer crossed the epithelium or remained in the lumen.

For biodistribution studies, CD1 mice were treated intravenously with PBS as negative control, or 1 mg/kg G2-S16-FITC dendrimer and sacrificed 30 min or 24 h after treatment In the experiments using 3 daily injections mice were sacrificed 24 h after receiving the last injection. Brain, heart, kidney, liver, and spleen tissues were extracted, washed with PBS and visualized using an In Vivo Imaging System (IVIS), (Lumina Image System, Caliper Life Sciences, Waltham, MA, USA) and displayed as an overlay image of light field and fluorescence images representing the intensity of the signal.

### 4.6. Histological Studies

BALB/c mice treated vaginally, as previously described, were collected and flash frozen in Tissue-Tek O.C.T. (Sakura Finetex, St. Torrance, CA, USA) in dry ice. Following cryogenic sectioning of the vagina, fornix and ectocervix, slides were fixed and permeabilized by incubation in 0.1% Triton X-100 for 5 min followed by a PBS wash. The samples were blocked for 30 min with 5% BSA, stained with anti-mouse CD192-Alexa Fluor^®^ 647 (Thermofishe, Alcobendas, Madrid, Spain) at a 1:100 dilutions in 2.5% BSA for 1 h, and washed 3 times with PBS. The samples were mounted with a ProLong Gold anti-fade reagent with DAPI (Thermo Fisher Scientific, Waltham, MA, USA). The slides were observed under the microscope and images were acquired with a Zeiss LSM710 confocal microscope using Zen 2011 software (Carl Zeiss Microimaging Inc., Thornwood, NY, USA).

The presence of histological lesions caused by the treatment with G2-S16 dendrimer was evaluated after hematoxylin-eosin staining. Tissue samples were dehydrated by passage using increasing concentrations of ethanol and two immersions in xylene (AnalaR (VWR) before being embedded in paraffin (Leica, Wetzlar, Germany). The samples were cut using a RM2145 semimotorized microtome (Leica, Wetzlar, Germany), and subsequently processed for staining. For dewaxing, samples were immersed for 10 min in two baths of xylene, followed by three baths of 5 min in solutions of a decreasing concentration of ethanol (100%, 90% and 70%). After staining by incubation with hematoxylin (Merck, Madrid, Spain) for 5 min, and eosin (Merck, Madrid, Spain) for another 5 min, the tissue samples were dehydrated by passage through increasing concentrations of ethanol (70%, 96%, and 100%) and two immersions in xylene, and mounted with DPX (Prolabo, Obregón, Mexico).

The histological lesions were evaluated in each sample as the presence of injury parameters specific to each tissue. A score was assigned to each parameter depending on the degree of the lesion observed as previously described [9].

The presence of histological lesions in the vaginal epithelium after G2-S16 vaginal application was studied after 7 days (BALB/c mice) or 14 days of treatment (BALB/c and CD1 mice). The treatment was performed as previously described. Histological damage was described as injury in the vaginal epithelium, inflammatory infiltrate, vascular congestion and/or oedema in the submucosa. The assigned values were added up and determined the level of vaginal irritation as minimal 1–4, average 5–8, moderate 9–11 and severe 12–16 [9].

In order to determine the systemic effects of high blood concentrations of G2-S16 dendrimer, brain, heart, kidney, liver, and spleen were studied in CD1 mice after 14 days of daily intravenous treatment with G2-S16 dendrimer (1 and 2.5 mg/kg) PBS as negative control.

### 4.7. Hemogram and Biochemical Blood Analysis

The CD1 mice were i.v. injected with either 1 mg/kg or 2.5 mg/kg for 7 consecutive days. At that time, blood was extracted by cardiac puncture. Blood was kept in 1 mL EDTA blood vials for the hemogram studies or with heparin for the biochemical analyses. Analysis were performed using automatic analyzers.

## 5. Conclusions

Taken together, the results indicate that the G2-S16, an effective nanoparticle that prevents HIV infection in vitro, is biocompatible and safe when administered well locally in the vagina or by intravenous injection in mice. This suggests that this molecule can be a used effectively alone, or in combination with an antiretroviral drug to prevent HIV infection.

## Figures and Tables

**Table 1:** Evaluation of tissue damage after 7 or 14 daily vaginal applications of G2-S16 dendrimer on BALB/c mice. The existence of injury in the vaginal epithelium was determined by histological evaluation after hematoxylin-eosin staining in each sample.

**7 CONSECUTIVE DAYS**	**PBS**		**N9 (8%)**			**G2-S16 (3%)**		**G2-S16 FITC (0.3%)**				**COMBINATION**			
Epithelial lesion	0 ^1^	0	2	3		1	1	1	0		3	1	2	1	
Inflammatory infiltrate	1	0	2	4		1	1	1	1		1	0	2	2	
Vascular congestion	0	0	2	2		1	1	0	1		1	1	1	1	
Edema/fibrosis	0	0	3	4		0	0	1	1		0	0	0	1	
TOTAL SCORE	1	0	9	13		3	3	3	3		5	2	5	5	
AVERAGE SCORE	0.5		11			3		4				4			
**14 CONSECUTIVE DAYS**	**PBS**					**N9 (8%)**					**G2-S16 (3%)**				
Epithelial lesion	1	0	0	0	2	3	3	3	4	4	1	0	0	1	1
Inflammatory infiltrate	2	0	2	1	0	4	2	3	2	2	2	1	1	0	1
Vascular congestion	0	2	2	2	2	3	3	4	3	3	1	1	0	1	1
Edema/fibrosis	0	0	0	0	0	3	3	3	2	2	0	0	0	0	0
TOTAL SCORE	2	2	4	3	4	13	11	13	11	11	4	2	1	2	3
AVERAGE SCORE	3.2					11.8					2.4				

^1^ These values were added up and determined the level of vaginal irritation as minimum 1–3, average 4–6, moderate 7–9 and severe >9+. PBS was applied as negative control and N9 as positive control for tissue damage.

**Table 2:** Evaluation of tissue damage. Results of CD1 mice after daily treatment with G2-S16 dendrimer for 14 consecutive days by vaginal application.

CD1 Mice	PBS					N9 (8%)					G2-S16 (3%)				
Epithelial lesion	0	0	0	0	2	4	2	3	2	1	1	1	3	2	2
Inflammatory infiltrate	0	0	0	0	0	1	3	1	1	2	1	1	0	1	0
Vascular congestion	0	2	1	0	0	2	1	2	2	3	0	2	1	1	0
Edema/fibrosis	0	0	0	0	0	1	3	3	0	2	0	2	1	0	0
TOTAL SCORE	0	2	1	0	2	8	9	9	5	8	2	6	5	4	2
AVERAGE SCORE			1					7.8					3.8		

These values were added up and determined the level of vaginal irritation as minimum 1–3, average 4–6, moderate 7–9 and severe >9+. PBS was applied as negative control and N9, as positive control of tissue damage.

## Data Availability

The data will be available from the corresponding author following reasonable request.

## Supplementary Materials

The following supporting information can be downloaded at: https://www.mdpi.com/article/10.3390/ijms23052565/s1.

## References

[1] Janaszewska A., Lazniewska J., Trzepinski P., Marcinkowska M., Klajnert-Maculewicz B. (2019). Cytotoxicity of Dendrimers. Biomolecules.

[2] Relano-Rodriguez I., Munoz-Fernandez M.A. (2020). Emergence of Nanotechnology to Fight HIV Sexual Transmission: The Trip of G2-S16 Polyanionic Carbosilane Dendrimer to Possible Pre-Clinical Trials. Int. J. Mol. Sci..

[3] Notario-Perez F., Ruiz-Caro R., Veiga-Ochoa M.D. (2017). Historical development of vaginal microbicides to prevent sexual transmission of HIV in women: From past failures to future hopes. Drug Des. Dev. Ther..

[4] Orza L., Bass E., Bell E., Crone E.T., Damji N., Dilmitis S., Tremlett L., Aidarus N., Stevenson J., Bensaid S. (2017). In Women’s Eyes: Key Barriers to Women’s Access to HIV Treatment and a Rights-Based Approach to their Sustained Well-Being. Health Hum. Rights.

[5] Phillips T.K., Teasdale C.A., Geller A., Ng’eno B., Mogoba P., Modi S., Abrams E.J. (2021). Approaches to transitioning women into and out of prevention of mother-to-child transmission of HIV services for continued ART: A systematic review. J. Int. AIDS Soc..

[6] Okutomi T., Minakawa S., Hirota R., Katagiri K., Morikawa Y. (2020). HIV Reactivation in Latently Infected Cells with Virological Synapse-Like Cell Contact. Viruses.

[7] Coutinho C., Sarmento B., das Neves J.J. (2017). Targeted microbicides for preventing sexual HIV transmission. J. Control. Release.

[8] Maciel D., Guerrero-Beltran C., Cena-Diez R., Tomas H., Munoz-Fernandez M.A., Rodrigues J. (2019). New anionic poly(alkylideneamine) dendrimers as microbicide agents against HIV-1 infection. Nanoscale.

[9] Chonco L., Pion M., Vacas E., Rasines B., Maly M., Serramia M.J., Lopez-Fernandez L., De la Mata J., Alvarez S., Gomez R. (2012). Carbosilane dendrimer nanotechnology outlines of the broad HIV blocker profile. J. Control. Release.

[10] Sepulveda-Crespo D., Cena-Diez R., Jimenezl J.L., Angeles Munoz-Fernandez M. (2017). Mechanistic Studies of Viral Entry: An Overview of Dendrimer-Based Microbicides As Entry Inhibitors against Both HIV and HSV-2 Overlapped Infections. Med. Res. Rev..

[11] Cena-Diez R., Garcia-Broncano P., Javier de la Mata F., Gomez R., Resino S., Munoz-Fernandez M. (2017). G2-S16 dendrimer as a candidate for a microbicide to prevent HIV-1 infection in women. Nanoscale.

[12] Briz V., Sepulveda-Crespo D., Diniz A.R., Borrego P., Rodes B., de la Mata F.J., Gomez R., Taveira N., Munoz-Fernandez M.A. (2015). Development of water-soluble polyanionic carbosilane dendrimers as novel and highly potent topical anti-HIV-2 microbicides. Nanoscale.

[13] Cena-Diez R., Garcia-Broncano P., de la Mata F.J., Gomez R., Munoz-Fernandez M.A. (2016). Efficacy of HIV antiviral polyanionic carbosilane dendrimer G2-S16 in the presence of semen. Int. J. Nanomed..

[14] Martin-Moreno A., Sepulveda-Crespo D., Serramia-Lobera M.J., Perise-Barrios A.J., Munoz-Fernandez M.A. (2019). G2-S16 dendrimer microbicide does not interfere with the vaginal immune system. J. Nanobiotechnol..

[15] Guerrero-Beltran C., Prieto A., Leal M., Jimenez J.L., Munoz-Fernandez M.A. (2019). Combination of G2-S16 dendrimer/dapivirine antiretroviral as a new HIV-1 microbicide. Future Med. Chem..

[16] Guerrero-Beltran C., Rodriguez-Izquierdo I., Serramia M.J., Araya-Duran I., Marquez-Miranda V., Gomez R., de la Mata F.J., Leal M., Gonzalez-Nilo F., Munoz-Fernandez M.A. (2018). Anionic Carbosilane Dendrimers Destabilize the GP120-CD4 Complex Blocking HIV-1 Entry and Cell to Cell Fusion. Bioconjug. Chem..

[17] Rodriguez-Izquierdo I., Natalia C., Garcia F., Los Angeles Munoz-Fernandez M. (2019). G2-S16 sulfonate dendrimer as new therapy for treatment failure in HIV-1 entry inhibitors. Nanomedicine.

[18] Sepulveda-Crespo D., Serramia M.J., Tager A.M., Vrbanac V., Gomez R., De La Mata F.J., Jimenez J.L., Munoz-Fernandez M.A. (2015). Prevention vaginally of HIV-1 transmission in humanized BLT mice and mode of antiviral action of polyanionic carbosilane dendrimer G2-S16. Nanomedicine.

[19] Gilbert H.N., Wyatt M.A., Pisarski E.E., Muwonge T.R., Heffron R., Katabira E.T., Celum C.L., Baeten J.M., Haberer J.E., Ware N.C.J. (2019). PrEP Discontinuation and Prevention-Effective Adherence: Experiences of PrEP Users in Ugandan HIV Serodiscordant Couples. Acquir. Immune Defic. Syndr..

[20] Straubinger T., Kay K., Bies R. (2019). Modeling HIV Pre-Exposure Prophylaxis. Front Pharmacol..

[21] Celum C., Baeten J. (2020). PrEP for HIV Prevention: Evidence, Global Scale-up, and Emerging Options. Cell Host Microbe.

[22] Roddy R.E., Zekeng L., Ryan K.A., Tamoufe U., Weir S.S., Wong E.L.N. (1998). A controlled trial of nonoxynol 9 film to reduce male-to-female transmission of sexually transmitted diseases. Engl. J. Med..

[23] Amaral E., Perdigao A., Souza M.H., Mauck C., Waller D., Zaneveld L., Faundes A. (2006). Vaginal safety after use of a bioadhesive, acid-buffering, microbicidal contraceptive gel (ACIDFORM) and a 2% nonoxynol-9 product. Contraception.

[24] Abdool Karim S.S., Richardson B.A., Ramjee G., Hoffman I.F., Chirenje Z.M., Taha T., Kapina M., Maslankowski L., Coletti A., Profy A. (2011). Safety and effectiveness of BufferGel and 0.5% PRO2000 gel for the prevention of HIV infection in women. AIDS.

[25] Pirrone V., Wigdahl B., Krebs F.C. (2011). The rise and fall of polyanionic inhibitors of the human immunodeficiency virus type 1. Antiviral Res..

[26] McCormack S., Ramjee G., Kamali A., Rees H., Crook A.M., Gafos M., Jentsch U., Pool R., Chisembele M., Kapiga S. (2010). PRO2000 vaginal gel for prevention of HIV-1 infection (Microbicides Development Programme 301): A phase 3, randomised, double-blind, parallel-group trial. Lancet.

[27] Stadler J., Scorgie F., van der Straten A., Saethre E. (2016). Adherence and the Lie in a HIV Prevention Clinical Trial. Med. Anthropol..

[28] Van der Straten A., Stadler J., Montgomery E., Hartmann M., Magazi B., Mathebula F., Schwartz K., Laborde N., Soto-Torres L. (2014). Women’s experiences with oral and vaginal pre-exposure prophylaxis: The VOICE-C qualitative study in Johannesburg, South Africa. PLoS ONE.

[29] Seidman D., Weber S., Aaron E. (2017). Dapivirine Vaginal Ring for HIV-1 Prevention. N. Engl. J. Med..

[30] Abdool Karim Q., Abdool Karim S.S., Frohlich J.A., Grobler A.C., Baxter C., Mansoor L.E., Kharsany A.B., Sibeko S., Mlisana K.P., Omar Z. (2010). Effectiveness and safety of tenofovir gel, an antiretroviral microbicide, for the prevention of HIV infection in women. Science.

[31] Balkus J.E., Palanee-Phillips T., Reddy K., Siva S., Harkoo I., Nakabiito C., Kintu K., Nair G., Chappell C., Kiweewa F.M. (2017). Brief Report: Dapivirine Vaginal Ring Use Does Not Diminish the Effectiveness of Hormonal Contraception. J. Acquir. Immune Defic. Syndr..

[32] Nel A., van Niekerk N., Kapiga S., Bekker L.G., Gama C., Gill K., Kamali A., Kotze P., Louw C., Mabude Z. (2016). Safety and Efficacy of a Dapivirine Vaginal Ring for HIV Prevention in Women. N. Engl. J. Med..

[33] Romano J., Variano B., Coplan P., Van Roey J., Douville K., Rosenberg Z., Temmerman M., Verstraelen H., Van Bortel L., Weyers S. (2009). Safety and availability of dapivirine (TMC120) delivered from an intravaginal ring. AIDS Res. Hum. Retrovir..

[34] Schader S.M., Colby-Germinario S.P., Schachter J.R., Xu H., Wainberg M.A. (2011). Synergy against drug-resistant HIV-1 with the microbicide antiretrovirals, dapivirine and tenofovir, in combination. AIDS.

[35] Baeten J.M., Palanee-Phillips T., Brown E.R., Schwartz K., Soto-Torres L.E., Govender V., Mgodi N.M., Matovu Kiweewa F., Nair G., Mhlanga F. (2016). Use of a Vaginal Ring Containing Dapivirine for HIV-1 Prevention in Women. N. Engl. J. Med..

[36] Else L.J., Taylor S., Back D.J., Khoo S.H. (2011). Pharmacokinetics of antiretroviral drugs in anatomical sanctuary sites: The male and female genital tract. Antivir. Ther..

[37] Bunge K.E., Levy L., Szydlo D.W., Zhang J., Gaur A.H., Reirden D., Mayer K.H., Futterman D., Hoesley C., Hillier S.L. (2020). Brief Report: Phase IIa Safety Study of a Vaginal Ring Containing Dapivirine in Adolescent Young Women. J. Acquir. Immune Defic. Syndr..

[38] Vanpouille C., Arakelyan A., Margolis L. (2012). Microbicides: Still a long road to success. Trends Microbiol..

[39] Zaias J., Mineau M., Cray C., Yoon D., Altman N.H.J. (2009). Reference values for serum proteins of common laboratory rodent strains. Am. Assoc. Lab. Anim. Sci..

[40] Rasines B., Sanchez-Nieves J., Maiolo M., Maly M., Chonco L., Jimenez J.L., Munoz-Fernández M.A., de la Mata F.J., Gomez R. (2012). Synthesis, structure and molecular modelling of anionic carbosilane dendrimers. Dalton Trans..

