# Supplementary material for: Safety of G2-S16 Polyanionic Carbosilane Dendrimer as Possible HIV-1 Vaginal Microbicide
Source: Int J Mol Sci. 2022 Feb 25;23(5):2565. doi: 10.3390/ijms23052565 (PMC8910216; doi:10.3390/ijms23052565)
Supplement: Supplementary file 1 [file ijms-23-02565-s001.zip › ijms-1560162-supplementary.pdf]

SUPPLEMENTARY INFORMATION

Table S1. Effect of daily intravenous treatment with G2-S16 dendrimer for 7 consecutive days. on CD1 mice hemogram. CD1 mice were treated with daily intravenous applications of either PBS or G2-S16 dendrimer (1 and 2.5 mg/Kg) for 7 consecutive days as indicated in Methods. Then, blood was drawn and the indicated parameters of the hemogram were determined. Data represent means ± s.e.m. of 3 (PBS) or 5 (G2-S16, 1 and 2.5 mg/mL) mice. [a] $p<0.05$ as compared with PBS-treated animals.

**Parameter**			
	**PBS**	**1 mg/kg**	**2.5 mg/kg**
**RBC (10^6/µL)**	8.34 ± 0.49	6.99 ± 2.71	8.82 ± 2.94
**Haematocrit (%)**	45.77 ± 3.3	51.15 ± 4.56	46.05 ± 15.36
**Haemoglobin(g/dL)**	14.40 ± 0.75	15.40 ± 2.1	14.87 ± 4.95
**MCV (fL)**	54.73 ± 0.78	50.77 ± 1.3	52.2 ± 17.40
**MCH (pg)**	17.35 ± 0.12	15.78 ± 0.95	16.85 ± 5.62
**MCHC (g/dL)**	31.53 ± 0.67	31.13 ± 1.83	32.25 ± 10.75
**RDW (%)**	21.03 ± 0.38	19.73 ± 2.43	22.05 ± 7.63
**WBC (10^3/µL)**	2.06 ± 0.50	1.55 ± 0.64	3.18 ± 1.06
**Platelets (10^3/µL)**	179.67 ± 94.17	464 ± 102.71[a]	215.5 ± 76.49
**PDW (fL)**	10.30 ± 3.43	8.53 ± 0.23	10.20 ± 3.4
**MPV (fL)**	8.10 ± 0.21	7.5 ± 0.40	9.03 ± 3.04
**Platelecrit (%)**	0.14 ± 0.07	0.35 ± 0.08[a]	0.20 ± 0.08

RBC, red blood cells; MCV, mean corpuscular volume; MCH, mean corpuscular hemoglobin; MCHC, mean corpuscular hemoglobin concentration; RCDW, red cell distribution wide; WBC, white blood cells, PDW, platelet distribution wide; MPV, mean platelet volume.

Table S2. Effect of daily intravenous treatment with G2-S16 dendrimer for 7 consecutive days. on CD1 mice plasma biochemical parameters. CD1 mice were treated with daily i.v. applications of either PBS or G2-S16 dendrimer (1 and 2.5 mg/Kg) for 7 consecutive days as indicated in Methods. Then, blood was drawn and the indicated plasma biochemical parameters were determined. Data represent means + s.e.m. of 3 (PBS) or 5 (G2-S16, 1 and 2.5 mg/mL) mice. a $p<0.05$ as compared with PBS-treated animals.

**Parameter**			
	**PBS**	**1 mg/kg**	**2.5 mg/kg**
**Glucose (mg/dL)**	57.33 ± 11.1	74.33 ± 6.25	70.33 ± 12.85
**Creatinine (mg/dL)**	0.15 ± 0.05	0.10 ± 0.03	0.10 ± 0.03
**Urea (mg/dL)**	21 ± 7.02	19.33 ± 1.26	18.33 ± 2.08
**Phosphorus (mg/dL)**	5.8 ± 1.95	5.93 ± 0.77	7.33 ± 0.63
**Lipase (U/L)**	852.00 ± 284.07	812.67 ± 43.07	869.33 ± 15.18
**ALT (U/L)**	165.2 ± 58.5	81 ± 3.04[a]	92.67 ± 12.25[a]
**ALKP (U/L)**	81.33 ± 39.54	81.67 ± 4.54	68.67 ± 12.58
**Bilirubin Total (mg/dL)**	1.65 ± 0.55	0.73 ± 0.08[a]	0.60 ± 0.09[a]
**Cholesterol (mg/dL)**	107.50 ± 36.25	122.67 ± 4.75	85.67 ± 5.13
**Protein Total (g/dL)**	6.67 ± 0.38	6.1 ± 0.36	6.5 ± 0.1
**Albumin (g/dL)**	3.40 ± 0.12	3.13 ± 0.13	3.03 ± 0.06
**Globulin (g/dL)**	3.27 ± 0.32	2.97 ± 0.46	3.5 ± 0.09
**Alb:Glob Ratio**	1.03 ± 0.09	1.2 ± 0.3	0.9 ± 0.05

ALT, alanine aminotransferase; ALKP, alkaline phosphatase.
